# Glances at the Hospitals

**Published:** 1901-08-17

**Authors:** 


### GLANCES AT THE HOSPITALS.

### WHITEHAVEN AND WEST CUMBERLAND INFIRMARY.

The income for the year 1899 amounted to £1,480, including in that sum a legacy of £215, and the expenditure reached £1,627; but some of this was spent on the building—for instance, the lift alone cost £160. The average cost per patient was £6 12s., the average cost per week per patient was 19s. 10½d., and the average cost per bed occupied was £53 10s. The total number of in-patients was 212; the average stay of each patient was 46 days, and the number of beds constantly in use was 26. The out-patients numbered 739, and the dispensary cases 371. The medical and surgical tables convey very little information: all that we are told concerning the operations is that 55 were performed during the year, that 48 were successful, that death followed in 3 cases, and that 4 were under treatment on December 31st, 1899.

### WEST BROMWICH HOSPITAL.

The total income for the year 1899 was £2,683, and the total expenditure was £2,746, but the latter sum included upwards of £170 spent on repairs and renewals, and the board of directors express pleasure that the financial condition of the hospital has improved. The building debt has been cleared off, and the deficiency on the working account has been reduced to £296. At the beginning of the year there were 44 in-patients, and 464 were admitted during the year, making a total of 508 under treatment. In addition to this number the eye department had 86 in-patients. The out-patients numbered 5,471, and the out-patients at the eye department were 2,462. The daily average number of in-patients in both departments was 44; the average stay in the hospital was 50 days; in the eye department the average stay was 24 days. The cost of the general in-patients was £4 10s. 5d. per head; in the eye department it was £2 19s. 1d. These figures show that the cost per bed occupied was £57 19s. 5d. An analytical table of diseases is given, but it has the common fault of ignoring the results of treatment. Even the table of operations does not say which were successful and which were not; nor is there any mention of the anæsthetic agent used in the hospital. The percentage of deaths after operation is given at 3·27, and a table shows the cause of death in all cases, but it is not always possible to extract from these the information required. We notice from the surgical tables that 11 cases of hernia were under treatment, from the table of operations that 9 were operated on, and from the table of deaths that 1 died; but we know not whether that death was one of the nine operated on, or one of the two not operated on.

### THE WEST NORFOLK HOSPITAL AT LYNN.

The total ordinary receipts were £2,335, and the total ordinary expenditure was £2,382. There was a balance of £511 in the treasurer's hands at the beginning of the year, and at the end of the year this had been reduced to £391. The average cost per bed occupied is not given, nor could we find any other statistics relative to the cost of maintenance of patients or staff. Including 25 patients in the hospital at the beginning of the year, there were 398 under treatment. Of these 320 were discharged cured or relieved, 11 were discharged for other reasons, 25 died, and 42 remained on the books on 31st December, 1899. The total number of out-patients was 796. Of these 559 were cured or relieved, 1 died, and 236 remained under treatment. In addition to the above numbers, 734 casualties were treated and 15 were sent to the Convalescent Home at Hunstanton. The medical and surgical statistics are extremely meagre. There is not even a table showing the number of operations performed; but we learn from a note that anæsthetics were administered 115 times. The medical and surgical tables merely name the diseases and give the number of patients who suffered from each disease. There were 21 cases of enteric fever, but we are not told how many of these died; and so on all through the list of diseases treated during the year.

